# Deoxynivalenol: A Major Player in the Multifaceted Response of *Fusarium* to Its Environment

**DOI:** 10.3390/toxins6010001

**Published:** 2013-12-19

**Authors:** Kris Audenaert, Adriaan Vanheule, Monica Höfte, Geert Haesaert

**Affiliations:** 1 Department of Applied BioSciences, Faculty Bioscience Engineering, Ghent University, Valentin Vaerwyckweg, 1, Ghent 9000, Belgium; E-Mails: adriaan.vanheule@ugent.be (A.V.); geert.haesaert@ugent.be (G.H.); 2 Department of Crop Protection, Laboratory of Phytopathology, Faculty Bioscience Engineering, Ghent University, Coupure links 653, Ghent 9000, Belgium; E-Mail: monica.hofte@ugent.be

**Keywords:** trichothecene, oxidative stress, virulence factor, fungicides, primary metabolism

## Abstract

The mycotoxin deoxynivalenol (DON), produced by several *Fusarium* spp., acts as a virulence factor and is essential for symptom development after initial wheat infection. Accumulating evidence shows that the production of this secondary metabolite can be triggered by diverse environmental and cellular signals, implying that it might have additional roles during the life cycle of the fungus. Here, we review data that position DON in the saprophytic fitness of *Fusarium*, in defense and in the primary C and N metabolism of the plant and the fungus. We combine the available information in speculative models on the role of DON throughout the interaction with the host, providing working hypotheses that await experimental validation. We also highlight the possible impact of control measures in the field on DON production and summarize the influence of abiotic factors during processing and storage of food and feed matrices. Altogether, we can conclude that DON is a very important compound for *Fusarium* to cope with a changing environment and to assure its growth, survival, and production of toxic metabolites in diverse situations.

## 1. Introduction

Fusarium head blight (FHB) is an important disease of small-grain cereals that is caused by a diverse set of *Fusarium* species. Although yield reduction is a serious consequence of *Fusarium* infection in the field, the primary interest in FHB research is driven mainly by the ability of *Fusarium* to produce mycotoxins that have toxic effects on plants, animals and humans [1,2]. Deoxynivalenol (DON) is one of the most prevalent mycotoxins encountered in grain fields. Consequently, although it is not the most toxic one, DON is considered to be the most economically important mycotoxin. DON belongs to the structural group of trichothecenes all bearing a common tricyclic 12,13-epoxytrichothec-9-ene core structure. Type A, B, C and D trichothecenes can be distinguished based on substitutions at position C-4, C-7, C-8 and/or C15 [3]. DON belongs to the type B trichothecenes and is mainly produced by *Fusarium graminearum* and *F. culmorum*, two important members of the FHB-causing species complex [4]. Historically, DON, also called vomitoxin, has been notorious because it provokes acute and chronic disease symptoms in humans and animals that consume contaminated grains [5]. Its toxic effects range from diarrhea, vomiting, gastro-intestinal inflammation, necrosis of the intestinal tract, the bone marrow and the lymphoid tissues. It causes inhibition of protein, DNA and RNA synthesis and inhibition of mitochondrial function. In addition, it has effects on cell division and membrane integrity and induces apoptosis [6]. Only after its toxicity for mammals had been established, were dedicated efforts initiated to unravel the conditions under which *Fusarium* species produce DON.

Many environmental factors are reported to affect DON levels during the infection process [7,8]. For instance, humidity and intensive rainfall during and after anthesis result in increased DON production and proliferated FHB symptoms [9,10,11,12,13,14,15,16]. Moreover, the weather conditions during the vegetative growth of wheat are important parameters determining *Fusarium* and DON load, reflecting the importance of survival of the primary inoculum present in soil and on crop debris during winter [14]. Furthermore, FHB and DON are influenced by many agronomic and other anthropogenic factors: no-, minimal-, or non-inversive tillage systems are beneficial for *Fusarium* [17]. Crop rotation, nitrogen fertilization, and weed management shape the structure of the soil biota and influence *Fusarium* survival [14,18,19]. Finally, the germplasm of the host has been shown to influence FHB and DON synthesis for example by the ability of resistant genotypes to metabolize DON [20,21].

Although this information is very valuable, in most studies no mechanistic clues are provided on how these factors affect the toxigenic machinery of the fungus. In addition, there are many other abiotic factors affecting DON of which the physiological relevance is not always clear. Obviously, a thorough insight into the functional rationale of DON production may provide hints towards an adjustment of control measures in order to avoid DON presence in the field. Therefore, we have placed the factors known to induce DON production in a relevant physiological frame, namely the different phases in the life cycle of *Fusarium* during the growing season of wheat (*Triticum* sp.) as a model host. Where possible we combine this information into working models that should be experimentally validated to obtain a holistic view on DON production by *Fusarium*.

## 2. The Saprophytic Phase

### 2.1. Survival of the Fittest

During the saprophytic phase, *F. graminearum* can survive on dead organic matter to persist in the absence of a living host, which is an important asset during the active invasion of hosts later on in the season. Therefore, saprophytic fitness is a significant component of the overall pathogen vigor [22]. Strikingly, information on the role of DON during this saprophytic period is scarce, although it covers a major part in the pathogen's life cycle and determines the primary inoculum load. Indeed, recently, DON production during the saprophytic survival on wheat stubble has been shown to be correlated with the aggressiveness of the isolates during their pathogenic phase [22].

The ability of most *F. graminearum* isolates to produce DON provides a dual advantage at the saprophytic state in the competition for niches on crop residues and organic matter. Firstly, DON is an antimicrobial metabolite that is effective against other eukaryotic soil organisms because of its interference with protein biosynthesis [5]. Secondly, DON can affect the metabolite production of other soil-residing fungi, such as *Trichoderma* sp., that are known for their strong outcompeting capacity by mycoparasitism, orchestrated by chitinases and other degrading enzymes [23]. In co-inoculation experiments, DON proved to repress the chitinase activity in *T. atroviride* [24], although a reduction in the *Trichoderma* biomass due to DON production by *F. graminearum* could not be observed [25].

Despite the very limited amount of information on the role of DON during the saprophytic phase, indirect evidence may come from comparative studies on the saprophytic survival of different *Fusarium* species. Apparently, *F. poae* which is considered a weak pathogen, is a better saprophytic survivor that outcompetes *F. graminearum* from soil and crop debris samples [26,27]. Since *F. poae* produces a more toxic blend of mycotoxins than *F. graminearum*, comprised of both type A and type B trichothecenes, it is tempting to speculate that this feature accounts for its better saprophytic survival capacity. The remarkable omnipresence of *F. poae* in the subsequent growth phase on living plant tissue, may thus originate from a “strength in numbers” strategy, originating from an inoculum build-up during the saprophytic phase.

### 2.2. Linkage between DON Production and Formation of Conidia and Ascospores

As the infection of *F. graminearum* is realized via production of conidia and ascospores, the formation of these reproductive structures is a very important phase in the pathogen’s life cycle. Recent research has shown that both DON production and conidia/ascospore formation are under tight regulation by overlapping cellular factors [28], some of which are mentioned below. APSES proteins are a conserved class of transcription factors regulating development, secondary metabolism and pathogenicity [29,30]. Recently, *FgStuA*, a *F. graminearum* gene encoding a protein with high homology to APSES transcription factors has been characterized. Using a knock-out approach, *FgStuA* was shown to influence spore development and DON biosynthesis amongst other processes [31]. Several other regulatory cellular proteins such as the C-type cyclin like protein CID1, the ZIF1 b-zip transcription factor and the Wor-1 like nuclear protein *Fg*p1 are all involved in sexual reproduction and influence DON production [32,33,34,35]. These results highlight a tight link between reproductive fungal development and secondary metabolite production. 

## 3. DON in the Pathogenic Phase: A Lethal Weapon of a Hemibiotrophic Cereal Killer

### 3.1. Plant Defense: A Matter of Making the Good Choices at the Right Time

Plants are endowed with a sophisticated set of plant defense mechanisms that can be activated upon pathogen infection. These defense responses can be divided into two main signaling pathways. One pathway involves a prompt induction of reactive oxygen species (ROS) followed by the accumulation of salicylic acid (SA), activating the plant’s defense machinery. This type of defense often coincides with a programmed cell death (PCD)-type response and a hypersensitive response (HR) that isolate the pathogen and deprive it from nutrients. This SA-type defense is generally accepted to be efficient against biotrophic pathogens that need viable cells for survival. The other pathway involves jasmonic acid (JA). This type of response is especially activated during the plant defense against necrotrophic pathogens [36,37].

However, some pathogens, such as DON-producing *Fusarium* spp, are hemibiotrophic and have both a biotrophic and a necrotrophic phase during the colonization of their host. Hence, in such interactions, a coordinated and ordered expression of SA- and JA-dependent defense responses in the plant is crucial to halt the fungus [38], but at the same time, it provides multiple opportunities for interference by the pathogen.

### 3.2. DON and the Plant Defense Response: Hijacking the Plants Oxidative Armor

**Figure 1:**
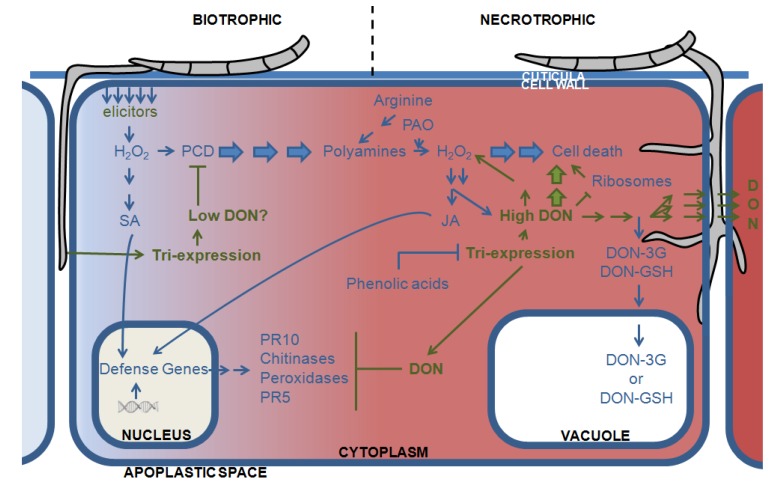
Hypothetical model of the effect of DON during the biotrophic and necrotrophic phases of *F. graminearum* infection of wheat, based on defense-related responses in wheat. The left part depicts the biotrophic phase and the right and red parts indicate the necrotrophic phase of the fungus. Green lines and arrows mark pathways of the fungus, whereas the blue lines reflect pathways of the plant. DON: deoxynivalenol; DON-3G: DON-glucoside; DON-GSH: DON-gluthatione; JA: jasmonic acid; PAO: polyamine oxidases; PCD: programmed cell death; PR: pathogenesis related; SA: salicylic acid; Tri: trichothecenes.

There is ample evidence suggesting that DON production during infection is a sophisticated strategy of the fungus to circumvent and hijack the plant’s defense system. When a rain-splashed conidium or wind-dispersed ascospore lands on the exposed vulnerable parts of a crop plant (glumae, floral cavity, lemma, palea, or anthers) during or just after anthesis, it can germinate and penetrate the plant [39]. An initial superficial and intercellular growth of the fungus is eventually followed by the actual penetration of the plant, which involves the formation of infection cushions and foot-like structures invaginating the host tissue [40,41]. In this first phase, the fungus grows biotrophically into the intercellular spaces and the role of DON is assumed to be unimportant. Still, during this biotrophic phase several reports describe *Tri* gene expression at the hyphal tip [42,43,44]. Recently, the ability of very low DON concentrations to inhibit PCD has been illustrated [45] which could interfere with PCD, thus disrupting the biotroph-type defense (Figure 1).

Afterward, the fungus switches to a more invasive intracellular growth, including necrosis and cell death [40]. During this second necrotrophic infection phase, the production of the mycotoxin DON becomes apparent and is necessary for the spread of the fungus in the rachis of wheat [46]. Previously, studies have demonstrated that *tri5* knockout mutants, which cannot produce DON because the inactive *Tri5* gene does not convert farnesyl pyrophosphate to trichodiene, are less virulent due to the lack of spread in the rachis, implying that DON is crucial in ear colonization [42,47,48]. 

The induction of cell death is a well-known defense strategy of plants against biotrophic but not against necrotrophic fungi [49]. In this context, it is interesting that high DON concentrations were shown to trigger H_2_O_2_ synthesis and subsequent cell death (Figure 1). Moreover, using an *in vitro* approach, several research groups demonstrated that H_2_O_2_ is an efficient inducer of DON production, especially when applied at early stages of spore germination [50,51,52]. Physiologically, these observations indicate that if H_2_O_2_ is one of the first defense molecules encountered by the invading *Fusarium* hyphae, it also establishes a positive feedback loop leading both to increased DON and H_2_O_2_ levels. Consequently, DON production by *Fusarium* in the necrotrophic infection phase may interfere with the two-step defense response against hemibiotrophs, because it directs the plant towards an oxidative burst which is not effective against necrotrophs. The eventual activation of H_2_O_2_-mediated defense responses comprising phenolic acids, chitinases, glucanases and peroxidases [46], might come too late or at the wrong time point for the plant to defend itself against the invasive necrotrophic growth of *F. graminearum*. Indeed, it is generally recognized that both timing and localization of defense or signaling compounds determine the outcome of a plant-pathogen interaction. The importance of H_2_O_2_ in the induction of DON was confirmed by the effectiveness of anti-oxidative phenolic acids, such as ferulic acid, to inhibit trichothecene accumulation at a transcriptional level *in vitro* [53,54,55]. In addition, *in planta*, the presence of ferulic acid in wheat cultivars correlated negatively with the accumulation of DON during *F. graminearum* infection [56].

Finally, it seems that DON-producing *Fusarium* species also interfere with the plant defense pathway further downstream of the oxidative burst. Indeed, SA can be used by *F. graminearum* as a carbon source [36], which may result in reduced expression of the typical SA-dependent defense genes such as pathogenesis-related protein 1 (PR1), nonexpressor of PR genes 1 (NPR1), and PR4, possibly impeding the control of symptoms development [36]. Moreover, the production of other defense-related compounds, such as PR10, chitinases, peroxidases, PR5, and PR10, is inhibited by DON at later time points during infection [49].

Nevertheless, DON is not essential in all *F. graminearum* plant interactions. For instance, although eventually a high DON load is measured as well, the infection of barley and rice with *F. graminearum* strains does not involve this mycotoxin [47,57,58].

### 3.3. Directing DON to the Vacuoles: Deprivation of the Pathogen of Its Virulence Factor

From the above, it is clear that DON is a powerful tool of *F. graminearum* to grow within the wheat host. Nevertheless, the plant is endowed with detoxification mechanisms to dampen the detrimental effects of the mycotoxin (for review [21]). Most important is the covalent binding of DON to hydrophilic molecules, such as glucose and glutathione (γ-glutamyl-cysteinyl-glycine, GSH). Conjugated DON is then transported via membrane-bound transporters to the vacuoles or apoplastic space [59,60]. The detoxifying effect of the conjugation is beyond dispute, but intriguingly, glutathione, a product derived from glyoxylate in the Calvin cycle, also plays an important role in modulating the redox status of the host cell, which determines the outcome of plant-pathogen interactions. Hence, it is tempting to speculate that through conjugation to DON, the fungus sequesters glutathione that affects the antioxidative status and, consequently, the defense machinery of the host cell. Still, it is important to notice that the oxidative status of plant cells is very complex with amongst others catalases, ascorbate peroxidases, superoxide dismutases and NADPH oxidases establishing the oxidative equilibrium. 

## 4. The Plant’s Primary Carbohydrate and Nitrogen Metabolism Feed into DON Production and Fungal Growth

Although current research particularly focuses on downstream defense signaling, the energy and carbon skeletons used in the defense reactions activated in wheat upon infection with *Fusarium* require the redistribution of energy from the primary metabolism of the plant. Interestingly, pathogens themselves seemingly also drain energy from the primary metabolism of the host to the advantage of their own pathogenic growth and production of their virulence factors [61,62].

When a plant is attacked by a pathogen, the availability of ready-to-use energy, reducing agents, and carbon skeletons is a prerequisite for optimal activation of defense. In many plant pathosystems, photosynthesis, which generates ATP and NADPH, decreases at the site of infection, establishing novel sink tissues [61]. Carbohydrate partitioning between source and sink tissues is a highly dynamic process during the plant’s life cycle and the physiological balance can easily be disrupted. Because of reduced photosynthesis, the plant will mobilize monosaccharides to the infection site by activating membrane-bound invertases that cleave apoplastic sucrose, thus generating energy and carbohydrate skeletons for diverse metabolic processes, including defense. However, sucrose is also an important inducer of the *Tri* gene machinery. Especially *Tri5* and *Tri4*, which are both involved in the initial steps of trichothecene biosynthesis by converting farnesylpyrophosphate to trichodiene and the latter to 15-decalonectrin, respectively, are strongly upregulated by sucrose, resulting in increased DON biosynthesis [63].

In the *F. graminearum*-wheat interaction, several plant invertases are upregulated, indicating that the fungus exploits sucrose not only as a trigger for DON biosynthesis, but also as a monosaccharide source that can be used for its own growth [64]. However, the contributions to the metabolism of the plant and of the fungus are difficult to distinguish. Indeed, pathogenic fungi also produce invertases that can potentially disturb the source-sink balance and the repartitioning of the carbon sources in the plant and, hence, affect the infection process.

The importance of nitrogen in plant defense is mainly situated at three levels. Firstly, nitrogen is indirectly involved as an energy source. Inorganic nitrogen is usually taken up as NH_4_ or NO_3_ after which it is incorporated into amino acids, such as glutamate, glutamine, asparagine, and aspartate via glutamine synthase. Subsequently, these amino acids are transported or stored in the plant by the glutamine-oxoglutarate aminotransferase (GOGAT) cycle. When the energy demand of the plant cells increases, for example upon pathogen infection, these amino acids are diverted to the energy-generating tricarboxylic acid (TCA) cycle, in part via the γ-aminobutyrate (GABA) shunt, leading to reducing equivalents and ultimately ATP [61,62]. Secondly, nitrogen is a main compound in the regulation of the redox status of plant cells. Reactive nitrogen species, such as nitric oxide (NO), but also polyamines, produced from the precursor l-arginine, can be directly involved in plant defense through HR induction [65]. Moreover, N-containing glutathione is an important antioxidant alleviating oxidative damage during an HR [66]. Thirdly, the plant’s nitrogen metabolism has been suggested to be involved in the defense response through a pivotal mechanism of evasion or endurance [62]. During the evasion process, implicated in a successful defense response against biotrophic pathogens, nitrogen is uploaded in the phloem as asparagine or glutamine and transported away from the invaded area to deprive the pathogen from the necessary nitrogen sources. During the endurance process nitrogen is remobilized from noninfected tissues providing infected cells with sufficient nitrogen to keep them alive; a strategy that is very efficient against necrotrophic pathogens [62].

**Figure 2:**
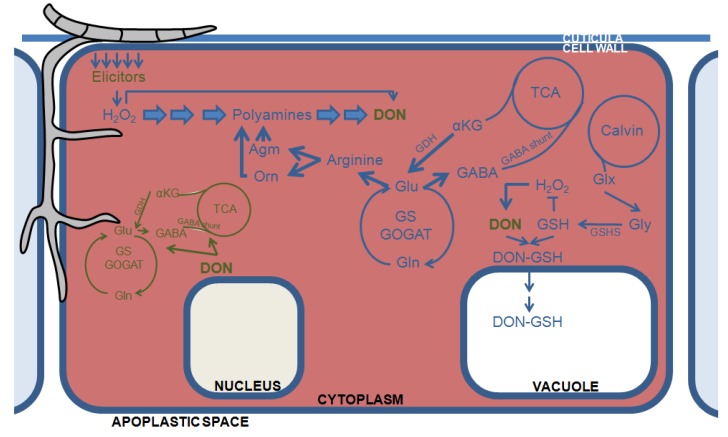
Hypothetical model of the interaction of DON with the primary metabolism of the host and the pathogen. Green lines and arrows indicate pathways of the fungus, blue lines reflect pathways of the plant. Bullet lines represent inhibitory actions. Agm: agmatine; αKG: α-ketoglutarate; DON: deoxynivalenol; GABA: γ-aminobutyric acid; GDH: glutamate dehydrogenase; Gln: glutamine; Glu: glutamate; Glx: glyoxylate; Gly: glycine; GOGAT: glutamine oxoglutarate aminotransferase; Orn: ornithine; TCA: tricarboxylic acid.

Just as with the carbohydrate metabolism, pathogens, including DON-producing *Fusarium* species, appear to hijack the primary nitrogen metabolism of the plant for their own benefit. For instance, several pathogens can use the plant’s amino acids as N-sources. Moreover, upon infection with *F. graminearum*, the primary GOGAT cycle appears to be redirected toward the production of ornithine and arginine, resulting in the formation of polyamines [67] (Figure 2). Indeed, a metabolo-proteomics approach revealed the induction of the agmatin-to-polyamine conversion [68]. As described above, the accumulation of polyamines can lead to ROS through the formation of NO and the action of polyamine oxidases [38,69], which could hypothetically contribute positively to the necrotrophic phase of *F. graminearum*. Finally, in an *in vitro* study, polyamines have been shown to induce DON production as well, further contributing to the fungus pathogenicity [70].

Although evidence is scarce, DON may interfere with aspects of the primary metabolism of the fungus itself. Although DON is considered to be a secondary metabolite, knocking out of the *Tri5* gene has a very profound impact on the primary metabolism of the fungus leading to decreased levels of glutamate and GABA and reduced glutamine synthase and GABA transferase activities [71]. Consequently the complete GABA shunt, TCA cycle, and polyamine metabolism are negatively affected (Figure 2). Conversely, upon infection, the GABA shunt becomes activated in DON-producing *F. graminearum* strains, suggesting a replenishment of the TCA cycle during the interaction with a host [72]. Moreover, metabolomic studies of wheat ears have revealed that the TCA cycle of the host is disturbed as well upon infection with *F. graminearum*, resulting in an increased activity of glutamate hydrogenase that converts α-ketoglutarate to glutamate although a direct link with DON production was not investigated. Interestingly, in other pathosystems involving necrotrophic and/or toxin-producing plant pathogens, a similar exhaustion of the TCA cycle of the host takes place, suggesting this might be a conserved and effective virulence strategy [73,74].

## 5. Of Crops and Men: The DON Molecule and Man’s Chemical Warfare

Because *Fusarium* infects an important economic crop cultivated within an agro-ecosystem, the plant-fungus interaction is more complex than in a natural ecosystem. Indeed, farmers interfere to minimize the presence of DON and other mycotoxins in the crop. Whereas the effect of chemical fungicides on fungal outgrowth is quite straightforward and generally results in reduced fungal load, reports on the impact of fungicides on the production of fungal secondary metabolites, especially mycotoxins, are rather inconsistent and fragmentary. Still, careful analysis of the information reveals important insights into the function of DON in the reaction of fungi to fungicide applications.

The effect of the strobilurin fungicide azoxystrobin on DON production varies from an increase [75,76,77,78] to a reduction [79] depending on environmental factors. Some other fungicides, such as carbendazim and thiram, have been tested for their efficiency to reduce DON in grain samples, but no clear effect was observed [80]. Nevertheless, the mycotoxin chemotype and the sensitivity toward carbendazim fungicides correlated well. As such, most strains producing nivalenol (NIV) or 15-acetyl-deoxynivalenol (15ADON) were susceptible, whereas all carbendazim-resistant isolates were 3-acetyl-deoxynivalenol (3ADON) producers [81].

**Figure 3:**
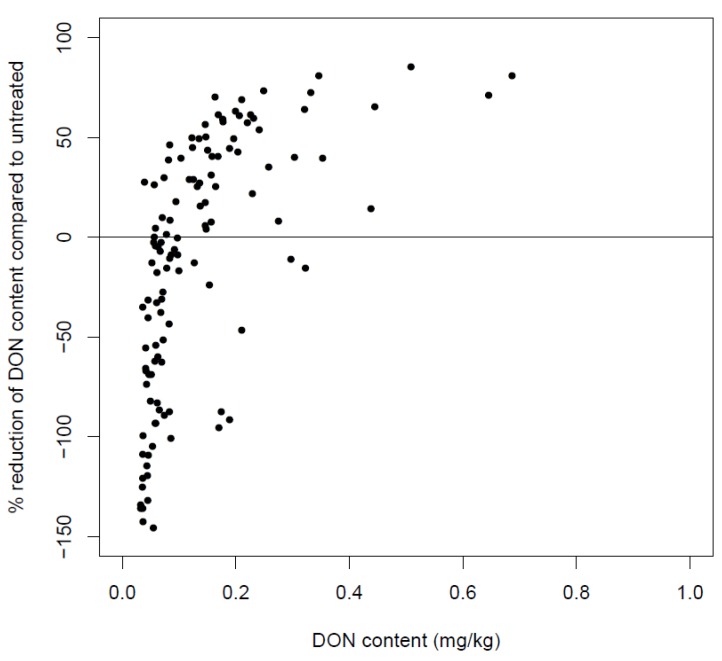
Percentage of reduced DON content after application of triazole fungicides at GS 39 and GS 55 on different wheat varieties in function of the DON content present in the untreated experimental field trials. All data points are the result of four independent replications and experiments were carried out at several locations in a three-year-experiment.

The most important fungicides currently used to control *Fusarium* are the azoles. A multi-year and multi-location experiment carried out in Belgium illustrated that the effect of azole fungicides with respect to DON depended on the DON concentration in the wheat host. In plants containing low and high DON amounts, fungicide applications often resulted in an increase and a reduction of DON load, respectively. These field trials also demonstrated that it was impossible to decrease the DON levels by more than 75% of the control fields (Figure 3). This observation may imply that highly contaminated fields, in which DON levels exceed the legislative values multiple fold, cannot be rescued by fungicide applications.

Within the group of azole fungicides, field doses of tebuconazole [75,77,82,83,84,85], metconazole [79,82,85], and prothioconazole [85] consistently reduced DON biosynthesis or content. In contrast, application of another azole fungicide, propiconazole, either decreased or increased DON levels [76,85]. Intriguingly, DON amounts are increased by application of a sublethal dose of prothioconazole, which is meticulously regulated through the production of H_2_O_2_ as an oxidative stress response of the fungus. Indeed, oxidative stress as a booster of toxigenic pathways is now considered a trait common to various toxigenic fungi from different genera of the fungal kingdom [86]. Moreover, qRT-PCR analyses have revealed that the expression of *Tri4*, *Tri5*, and *Tri11* was higher in cultures of *F. graminearum* isolates supplemented with sublethal concentrations of tebuconazole and propioconazole than that in nontreated controls, although the fold change in the *Tri* transcript levels differed according to the type of azole used [87].

Typically, azole sensitivity in fungi is modified by either point mutations in the cytochrome P450 monooxygenase-encoding target gene *CYP51* [88,89], overexpression of *CYP51* [90], presence of paralogous CYP51 genes [91], the presence of fungal drug transporters, belonging to the ABC or MDR classes [92], or an altered composition of the sterol content [93]. However, considering the effect of low fungicide levels on DON production, the question arises whether DON interferes with the fungicide effectiveness. Indirect proof comes from *in vitro* fungicide assays with a *tri5* knockout mutant of *F. graminearum*. The overall fitness and fecundity of the mutant was comparable to that of the parent strain; but, when homeopathic levels of azole fungicides were applied, only the mutant fungus promptly stopped growing [94]. Apparently, when a strain cannot produce its toxic secondary metabolite DON, it becomes hypersensitive to azole fungicides. Additionally, when *F. graminearum* strains were allowed to adapt to azole fungicides, they showed an increased production of the B-type trichothecene NIV [95].

Although direct evidence on the role of DON in fungicide resistance is currently lacking, *Sacharomyces cerevisiae* is known to be very resistant against DON because of the presence of multiple ABC transporters that pump the mycotoxin out of the cell [96]. In addition, expression of ABC transporters of the plant pathogen *Mycosphaerella graminicola* in an ABC transporter-lacking mutant of *S. cerevisiae* clearly indicated a wide functional overlap between the ABC transporters induced by azole fungicides and those by the A-type trichothecene diacetoxyscirpenol [97]. Finally, transcriptional profiling of ABC transporters upon fungicide application points toward a mechanism alleviating the impact of the fungicide [95]. Together, this fragmentary information seems to imply that the mycotoxin production capacity and resistance against fungicides converge at the ABC efflux level or other MDR pumps. It is not unlikely that (some) efflux pumps activated upon mycotoxin biosynthesis are also activated during exposure to fungicides. Interestingly, at least one ABC transporter has been shown to be important in the virulence of *F. graminearum*, but an effect on the mycotoxin efflux from the deletion mutant was not reported [98].

## 6. Abiotic Factors Influencing DON Biosynthesis in the Field and during Storage

The impact of abiotic factors on mycotoxin production has recently been reviewed [7,8]. Therefore, we highlight only new research findings that deal with environmental effects on DON biosynthesis.

### 6.1. pH

Although it is currently unknown whether and how the pH fluctuates during a wheat infection with *Fusarium*, it is well established that a low extracellular pH results in an increased trichothecene production [99]. *Tri* gene expression is regulated by a zinc finger transcription factor *Fg*Pac1 at acidic pH values [100], but the regulation at neutral or basic pH remains unclear. As information on the extracellular pH during wheat infection by *F. graminearum* and during grain storage remains scarce, it is very difficult to place the results on the pH effects in a physiological context. Probably, a dynamic window of pH fluxes influences DON production during the infection process.

### 6.2. a_w_ and Temperature

The availability of free water (a_w_) and the incubation temperature will determine whether there will be an outgrowth of *F. graminearum*, especially during storage of wheat grains after harvest. In addition, the toxigenic outcome of fungal growth also depends on the a_w_ value and the temperature. Indeed, high a_w_ values increase DON production in contaminated wheat grain batches [101] as well as elevating the incubation temperature from 15 °C to 30 °C [102]. Several reports also describe a clear interaction between temperature and a_w_ value [103].

### 6.3. Light

In plants, several important pathways follow a diurnal regulation based on the day/night regime. Although fungi do not depend on photosynthesis for their energy supply, their secondary metabolism is often fine-tuned by light. One of the most important light-regulatory protein complexes is the velvet complex, comprising at least *Fg*Ve1 (VeA) and *Fg*VeB. Although the velvet complex has been elaborately investigated with regard to the switch between asexual and sexual phases of the fungus, recent research highlights its significance in the regulation of the *Tri* gene machinery. By means of a gene replacement strategy, VeA has been demonstrated to regulate trichothecene production at the level of the biosynthetic genes *Tri4* and *Tri5* and the transcriptional regulator genes *Tri6* and *Tri10* [104,105]. Results with knockout mutants have revealed that *Fg*VeB plays a role in the regulation of *Tri5* and *Tri6* as well [106].

### 6.4. Post-Harvest Anthropogenic Factors Influencing the DON Content

After harvest, grains are often stored for some time in silos before final use as animal feed or human food. Although DON production during storage is, exceptions notwithstanding, rather rare, effects of changed storage conditions on fungal outgrowth and DON production have been reported. Modified storage atmosphere, chemical preservation systems, and biocontrol with lactic acid bacteria have been proposed as antifungal measures [102]. Detailed insights into the effect of these measures on DON production are still lacking. Chemical compounds, such as antioxidants and essential oils applied during storage of wheat grains clearly have a very variable impact on the DON levels. In an experiment in which wheat grains were inoculated with *F. graminearum* and subsequently treated with neutralized electrolyzed water, the ROS present in the electrolyzed water reduced the fungal load in the wheat commodities. However, at sublethal levels, this decrease in biomass coincided with an increase in DON level. The ROS liberated from the electrolyzed water oxidatively stimulated the *Tri* gene machinery to produce DON [94].

## 7. Conclusions and Challenges for the Future

In the present review, we gathered available data on diverse factors known to affect DON production by *Fusarium*. We combined this information into hypothetical models on the effect of DON on defense-related processes and the primary metabolism of wheat as a model host. Altogether, based on the present literature, we claim that DON is a molecule that is crucial throughout the fungal life cycle. During saprophytic survival, DON might be involved in competition for niche. Furthermore, DON production and conidia- and/or ascospore formation are tightly linked processes. 

During the interaction with its host, it seems that *Fusarium* uses DON to disturb the defense system at several critical time points of infection assuring successful colonization and symptom development (Figure 1). Moreover, DON appears to be deployed to hijack the primary C and N metabolism of the plant to improve fungal growth and production of virulence factors (Figure 2). Although parts of the proposed models are still highly speculative and not supported by direct experimental evidence, we hope they provide valuable working hypotheses for future research.

An additional challenge is to decipher the function of other type A and type B trichothecenes produced by other members of the FHB disease complex. Is the importance of DON in the life cycle of *Fusarium* spp. unique or can the functions be extrapolated to other mycotoxins? More generally, searching for parallels between *Fusarium* and other toxin-producing plant pathogens might reveal conserved infection strategies typical for this type of phytopathogens.
